# Molecular Characterization of *RXR* (Retinoid X Receptor) Gene Isoforms from the Bivalve Species *Chlamys farreri*

**DOI:** 10.1371/journal.pone.0074290

**Published:** 2013-09-16

**Authors:** Jia Lv, Liying Feng, Zhenmin Bao, Huihui Guo, Yueyue Zhang, Wenqian Jiao, Lingling Zhang, Shi Wang, Yan He, Xiaoli Hu

**Affiliations:** Key Laboratory of Marine Genetics and Breeding (MGB), Ministry of Education, College of Marine Life Sciences, Ocean University of China, Qingdao, China; University of Geneva, Switzerland

## Abstract

**Background:**

Bivalves are among the oldest classes of invertebrates, and they exhibit diverse types of sexual patterning. However, our current understanding of the mechanisms of sex determination and differentiation in bivalves remains very limited. The retinoid X receptors (RXRs), which are members of the nuclear receptor family, are involved in sex differentiation in many organisms.

**Results:**

In the present study, four full-length RXR-encoding cDNAs (*CfRXR*s) named *CfRXRa*, *CfRXRb*, *CfRXRc* and *CfRXRd* were retrieved from Zhikong scallop (*Chlamys farreri*). The four RXRs exhibited the conserved five-domain structure of nuclear receptor superfamily members and differed from each other only in the T-box of the C domain. The three variants, designated T (+4), T (+20) and T (+24), contained insertions of 4, 20 and 24 amino acids, respectively. The entire *CfRXR* gene is composed of eight exons and seven introns, and the four isoforms are generated *via* alternative mRNA splicing. Expression analysis showed that all four isoforms were expressed in both the testis and the ovary during the differentiation stage, whereas no expression was detected in the growth, mature or resting stages. This result suggests that *CfRXRs* are involved in germ cell differentiation in both sexes. The expression of the four isoforms was also detected in other tissues examined, including mantle, gill, digestive gland, and adductor muscle of sexually mature male and female Zhikong scallops, implying the multiple biological functions of *CfRXRs*.

**Conclusion:**

Our study presents the first report of RXR isoforms in bivalves. Further investigation of the functional roles of different RXR isoforms may provide deep insights into the regulatory mechanism of sex differentiation in *C* *. farreri* .

## Introduction

The Retinoid X receptor (RXR) superfamily of hormone nuclear receptors (NRs) has been implicated in several physiological and metabolic processes in vertebrates including growth, differentiation, reproduction, and apoptosis [1,2,3]. RXR functions as a transcription factor by forming a homodimer with itself or a heterodimer with another NR and then binding to direct repeats (DRs) of specific DNA sequence spaced by one to five nucleotides (DR1-DR5) in the promoter regions of the targeted genes [4]. Like other NRs, RXR consists of up to six domains (A-F): the A/B domain contains a ligand-independent transcriptional activation domain and varies in sequence and length; the C domain serves as the DNA-binding domain (DBD), in which a T-box interacts with a zinc finger on the RXR partner to provide the DBD dimerization interface [4]; the D domain is the hinge region and links the DBD to the E domain which is a ligand-binding domain (LBD); the F domain is another highly variable region that has not yet been functionally characterized in RXRs [5]. Vertebrate RXR proteins are encoded by three genes: *RXRα*, *RXRβ* and *RXRγ*. Each of the three genes can produce different RXR isoforms through the use of alternative promoters, splice sites or polyadenylation signals [6].

RXR homologs have also been characterized in invertebrates such as sponges [7], insects [8], crustaceans [9] and gastropods [10,11]. In insects, the differences among the RXR orthologs, known as ultraspiracle (USP) isoforms, appear to be centered in the A/B domain [12,13], similar to vertebrate RXRs [14]. Unlike insect RXRs, crustacean RXR isoforms contain different T-box [9,15] or LBD sequences [16,17]. The gastropod RXR isoforms that have been identified in *Nucella* *lapillus* and *Thais* *clavigera* contain insertions at the same position of the T-box [11,18].

Recent work has implicated the important role of RXRs in germ cell differentiation in invertebrates. In arthropods, USPs bind to the key sex determinant, methyl farnesoate, with high affinity [19]. *RXR* gene has been cloned from a green crab, and its expression profile in the ovary suggests that it plays a role in female reproduction [9]. Sexual differentiation in gastropods is currently of particular interest because organotin compounds have been shown to induce the development of male accessory sex organs in females. Recent findings support the hypothesis that this phenomenon is mediated through an RXR signaling pathway [10,18,20]. In addition, mud snail *RXR* mRNA levels increase in concert with differentiation in both sexes [21].

Marine bivalves exhibit different types of sexual patterning. For example, some scallops are dioecious, whereas others are simultaneous hermaphrodites with a few as protoandrous hermaphrodites (male when young, then later become female) or proterogynous hermaphrodites (female when young, then later become male). It is generally accepted that bivalves have no great difficulty transitioning from hermaphroditism to gonochorism and *vice versa* [22]. Meanwhile, the natural bivalve hermaphrodites have two regionally separate gonads, an ovary and a testis, whereas species with partial or occasional hermaphroditism have mosaic gonads. Because of the wide variations in the expression of sexuality, the bivalve mollusk represents an excellent animal model for the study of sex differentiation.

In this work, we cloned the *RXR* genes from Zhikong scallop (*Chlamys farreri*), a dioecious bivalve that is also an economically important aquaculture species in China, as an initial effort to study genes potentially involved in scallop reproduction. Four *RXR* isoforms (*CfRXR*s) generated from alternative mRNA splicing were obtained, and their expressions in the development cycle of the gonads for both sexes and in other tissues were analyzed. This is the first report of RXR isoforms in bivalves, and our results suggest potential roles for *CfRXRs* in germ cell differentiation in scallops.

## Materials and Methods

### Animals

Zhikong scallops (*Chlamys farreri*) were collected every month from Xunshan Hatchery Company in Shandong, China, over the course of one year, to obtain specimens with gonads from the differentiating stage to the resting stage. The gonads were collected from healthy individuals, frozen in liquid nitrogen and stored at -80°C. The reproductive status of the gonads was determined by histology. Other tissues, including mantle, gill, digestive gland, and adductor muscle of sexually mature male and female individuals were dissected and preserved as described above.

### Cloning and characterization of CfRXR

Total RNA from the male and female gonad tissues of eight scallops at four different stages of reproduction (differentiating, growth, mature and resting) was extracted using guanidinium isothiocyanate, as described by Hu et al. [23]. The RNA samples from the eight scallops were treated with DNase I (Takara Bio) and pooled together in equal quantities. The first-strand cDNA was synthesized from 2 µg of pooled total RNA using oligo(dT)_12-18_ and MMLV reverse transcriptase (Promega) in a final volume of 25 µl. A pair of degenerate primers (F1: 5'-TAYNTGYGARGGNTGYAARGG-3'; R1: 5'-TCYTGNACNGCYTCNCKYTTCAT-3'; R=A,G; Y=C,T; K=G,T; N=A,T,C, G) was designed based on the DNA binding and hinge domains of RXR and several homologous proteins, and these primers were used to amplify a 167-bp *RXR* cDNA fragment from the Zhikong scallop gonad tissue. PCR was carried out for 35 cycles of 94°C (30 s), 50°C (30 s), and 72°C (30 s) using a PTC-Peltier thermal cycler. The cDNA fragments were gel purified with a TIANgel Midi Purification Kit (TIANGEN), inserted into the pMD18-T vector (Takara Bio), and transformed into *E. coli* Top10 cells. Recombinant plasmids containing inserts were sequenced using an ABI 3730 sequencer. Then 5’- and 3’-rapid amplification of cDNA ends (RACE) was performed to obtain the 5’ and 3’ cDNA ends of *RXR* using the SMART RACE Kit (Clontech) according to the manufacturer’s instructions. Gene-specific primers 5'-CGGAAGGACCTTACTTATGCTTGCAGAG-3' and 5'-CTCTGCATCCAGAACCTTTTCAACAGGC-3' were used for 3’ RACE and 5’ RACE, respectively. The RACE products were purified, cloned and sequenced as above. Four *RXR* sequences were obtained, and named *CfRXRa, CfRXRb, CfRXRc* and *CfRXRd*, respectively. The entire cDNA sequences of *CfRXR*s were used to conduct a BLAST homology analysis using the genome sequence database of the Zhikong scallop, which has been sequenced at a coverage of 50× (unpublished data). The gene structure was characterized by comparing the cDNA sequences to the genomic sequence. The intron-exon boundaries were identified manually.

### Sequence analysis

Using DNAstar version 6.13, the full protein sequence of CfRXRa was aligned with the RXR sequences of other species obtained from NCBI’s GenBank database. Phylogenetic analysis was conducted using MAGA version 5.1. A phylogenetic tree was constructed using the neighbor-joining method based on the sequences of DNA-binding domain (DBD) and ligand-binding domain (LBD), respectively. The reliability of the branches was evaluated by bootstrapping with 1000 replicates.

### Expression analysis of CfRXRs

Reverse transcription PCR (RT-PCR) was perform to detect the expression levels of *CfRXRs*. Total RNA was extracted from the ovary and testis at the four stages of gonadal development, and other tissues (mantle, gill, digestive gland, and adductor muscle) of sexually mature male and female individuals. For each of these tissues, three scallops were used in the following gene expression analysis. The first-strand cDNA was synthesized as described above. A pair of gene-specific primers (F2: 5'-CGGAAGGACCTTACTTATGCTTGCAGAG-3'; R2: 5'-CTCTGCATCCAGAACCTTTTCAACAGGC-3'), flanking the variable T-box regions, was used to examine the expression levels of all the four *CfRXR* isoforms in gonads. The PCR products from different *CfRXR* isoforms were discriminated by length. Two *β-actin* primers (AF: 5'-CAATCTACGAAGGTTATGCC-3'; AR: 5'-CCTGTTCAAAGTCAAGTGC-3') were used to amplify a 186-bp *β-actin* gene fragment as an internal control. PCR was performed in a total volume of 20 µl containing 1 µl of cDNA, 0.2 mM of each dNTP, 0.2 mM of each primer (either F2 and R2 or AF and AR), 1.5 mM MgCl_2_, 1× buffer and 0.5 U Taq DNA polymerase (Takara Bio). The *CfRXRs* gene fragments were amplified for 30 cycles at 94°C for 1 min, 60°C for 1 min and 72°C for 0.5 min with a final extension step of 72°C for 5 min. Amplification of the *β-actin* gene fragment was performed as described above except that the number of cycles used was 25. The PCR products obtained from each tissue sample were separated on a 10% polyacrylamide gel, and the bands were detected using ethidium bromide staining.

## Results

### cDNA sequences and gene structures of CfRXRs

**Figure 1:**
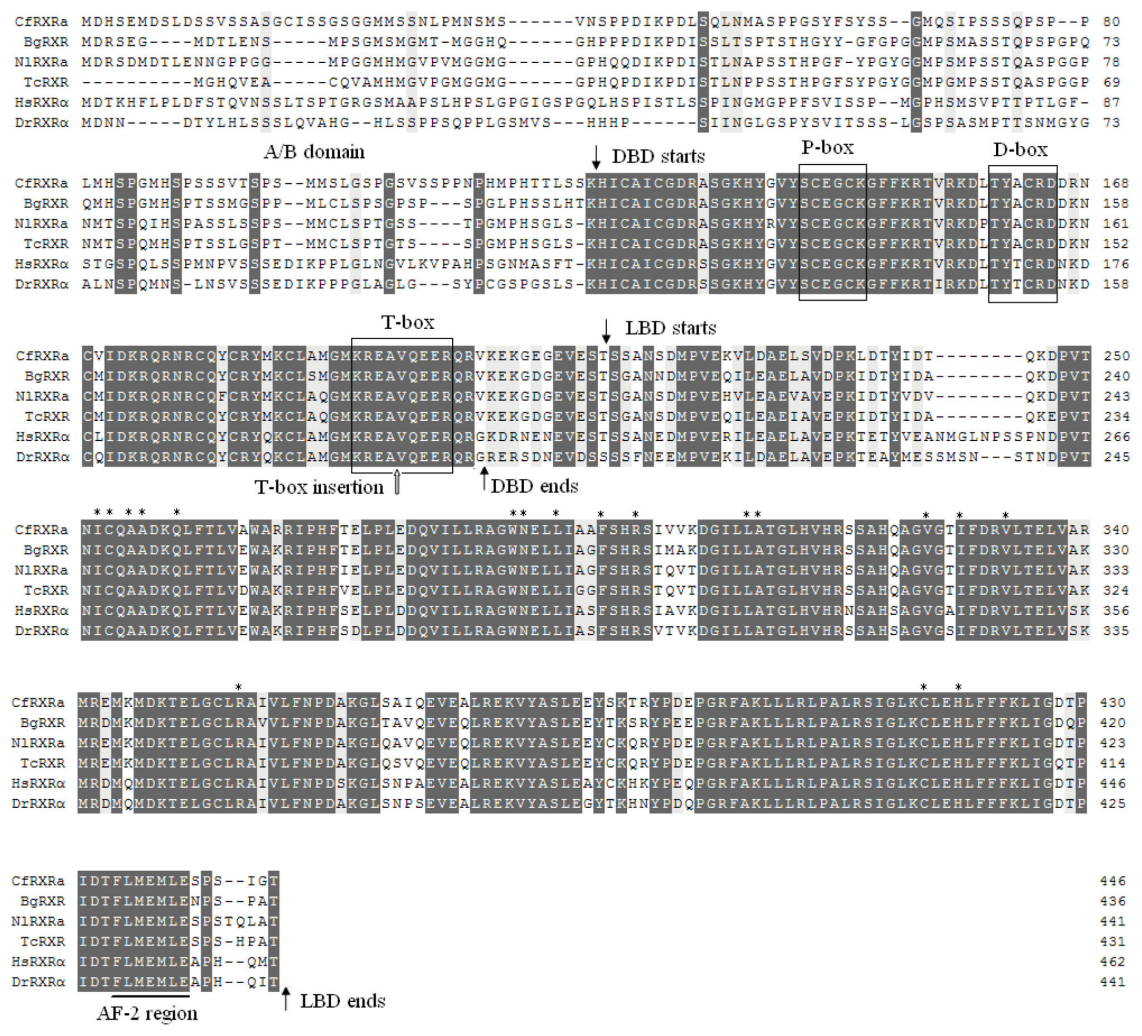
Comparison of the full-length amino acid sequence of CfRXRa with RXRs from *B* *. glabrata* (BgRXR, GenBank ID: AAL86461), *N* *. lapillus* (NlRXR, GenBank ID: ABS70715), *R* *. clavigera* (TcRXR; GenBank ID: AAU12572), *D. rerio* (DrRXRα, GenBank ID: AAC59720) and *H. sapiens* (HsRXRα; GenBank ID:NP_002948). The black-headed arrows represent the boundaries of functional domains and indicate the variable A/B domain and the conserved DBD and LBD regions. The positions of the T-box insertions in scallop and snail RXRs are indicated by hollow arrows. The P-box, D-box and T-box are highlighted with boxes. The AF2 region is underlined. The residues known to interact with 9-cis RA are indicated by an asterisk above the sequence text. Identical residues are shaded in dark grey, and similar residues are in light grey. The hyphens (-) indicate gaps that were inserted to maximize sequence similarity.

**Figure 2:**
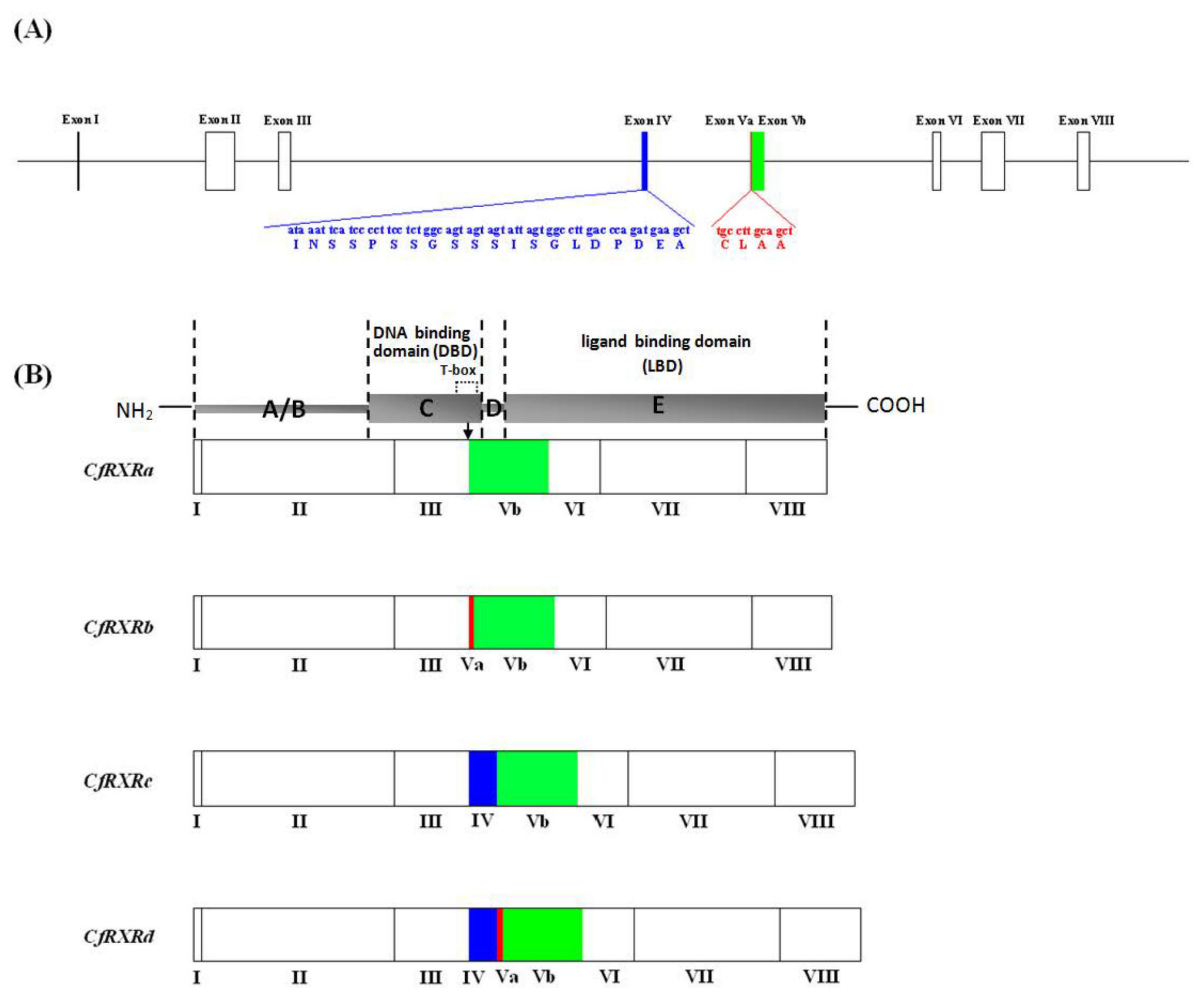
A schematic diagram showing the genomic structure and alternative splicing of the *CfRXRs* (to scale). Exons IV, Va and Vb are shown in blue, green and yellow, respectively. (A) Exons and introns are represented by boxes and lines, respectively. Exon IV is 60 bp and encodes for 20 amino acids. The 12-bp insertion encoding 4 amino acids is located in the exon Va. (B) Four alternatively spliced mRNAs corresponding to the four *CfRXR* isoforms. The arrow indicates the location of the insertion/deletions which are in the T-box of the C domain.

Four variant sequences were obtained by 3’ RACE, and then were verified by 5’ RACE. Four full-length cDNAs were obtained by piecing the 3’ and 5’ RACE sequences, which all encoded *C* *. farreri* RXR orthologs, and were named *CfRXRa, CfRXRb, CfRXRc and CfRXRd*, respectively. The four *CfRXR* isoforms were identical except for amino acid insertions/deletions located in the T-box of the C domain (Figure 1, Figure 2). The shortest gene isoform (*CfRXRa*) was 3051 bp in length and contained an ORF encoding 446 amino acids. The three variants, termed T (+4), T (+20) and T (+24) contained insertions of 4, 20 and 24 amino acids, respectively. Amino acid sequence comparisons revealed that the CfRXRs had all the main features typical of the RXR family (Figure 1). In CfRXRs, the P-box and D-box, the regions of the DBD that are important in controlling DNA recognition, were identical to those of other RXRs (Figure 1). The AF2 region is critical for ligand-dependent transactivation of RXR, and it was identical among the analyzed RXRs from both vertebrate and invertebrate (Figure 1). Residues that are known to interact with 9-cis retinoic acid (9-cis RA) in humans were identical among these species, too (Figure 1). The cDNA and deduced protein sequences of *CfRXRa, CfRXRb, CfRXRc* and *CfRXRd* have been deposited in the GenBank database with the accession numbers JQ778315, JQ778316, JQ778317 and JQ778318, respectively.

A BLAST search on the 50× coverage genome sequence database of the Zhikong scallop returned only one scaffold match. This suggests that there is only a single copy of *RXR* gene in the Zhikong scallop genome and that the four isoforms are generated *via* alternative mRNA splicing. Figure 2 shows the gene structure of *CfRXR*. Unlike human *RXR* genes, which contain ten exons [4], the *CfRXR* gene is composed of eight exons and seven introns (Figure 2A). The T-box in CfRXR spans three exons (exon III, IV and V), and the differences among CfRXR isoforms are in exons IV and V (Figure 2B).

### Phylogenetic analysis

**Figure 3:**
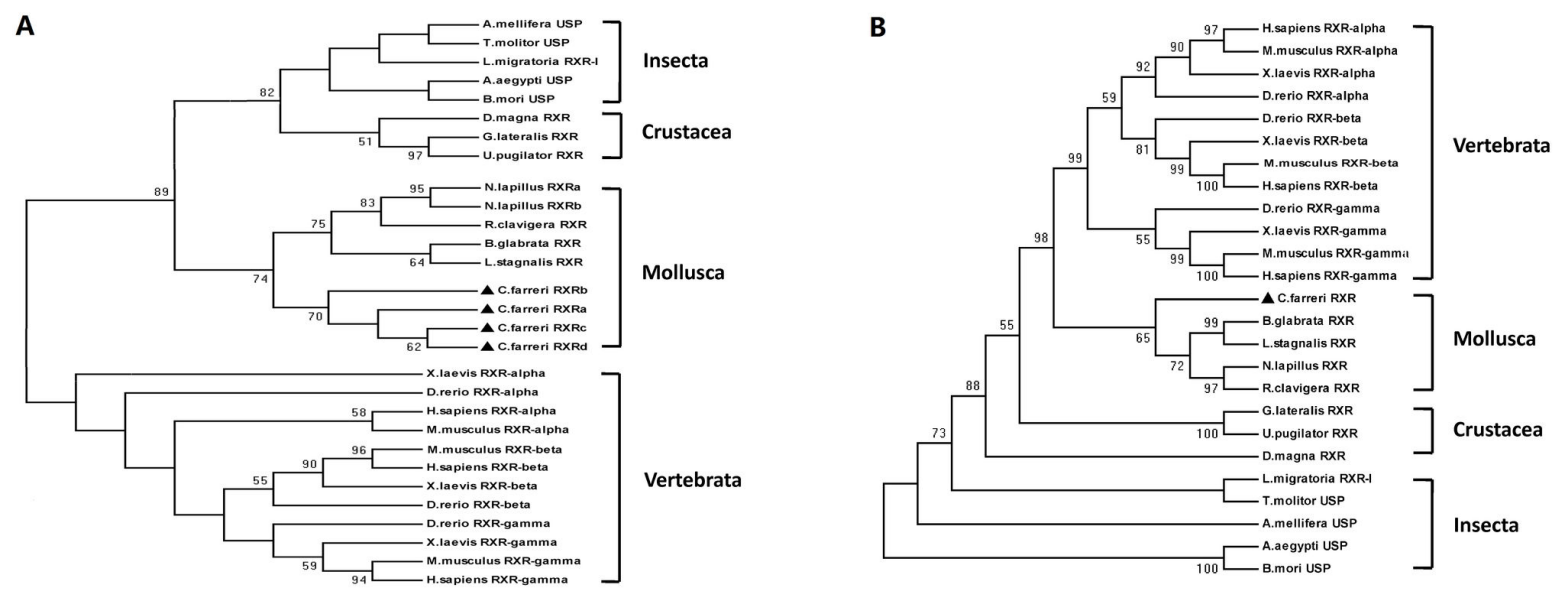
Phylogenetic analysis of RXR/USP. The tree was constructed using the neighbor-joining method based on the amino acid sequences of RXR/USP DBDs (A) and LBDs (B). The *C. farreri* RXR sequence is marked with black triangle, and the numbers at the nodes correspond to the bootstrap support expressed as the percentage of 1000 replicates. The Genbank accession numbers used to construct phylogenetic tree are as follows: *A. aegypti* USP (GenBank ID: AAG24886); *A. mellifera* USP (GenBank ID: AAP33487); *B. glabrata* RXR (GenBank ID: AAL86461); *B. mori* USP (GenBank ID:NP_001037470); *U. pugilator* RXR (GenBank ID: AAC32789); *D* *. magna* RXR (GenBank ID: ABF74729); *D. rerio* RXR-alpha (GenBank ID:NP_571228); *D. rerio* RXR-beta (GenBank ID:NP_571313); *D. rerio* RXR-gamma (GenBank ID:NP_001002345); *G. lateralis* RXR (GenBank ID: AAZ20368); *H. sapiens* RXR-alpha (GenBank ID: ABB96254); *H. sapiens* RXR-beta (GenBank ID: AAA60293); *H. sapiens* RXR-gamma (GenBank ID: AAA80681); *L. migratoria* RXR-l (GenBank ID: AAQ55293); *L. stagnalis* RXR (GenBank ID: AAW34268); *M. musculus* RXR-alpha (GenBank ID:NP_035435); *N.lapillus* RXRα (GenBank ID: ABS70715); *N. lapillus* RXRβ (GenBank ID: ABS70716); *R. clavigera* RXR (GenBank ID: AAU12572); *T. molitor* USP (GenBank ID: CAB75361); *X. laevis* RXR-alpha (GenBank ID: P51128); *X. laevis* RXR-beta (GenBank ID:NP_001080936); *X. laevis* RXR-gamma (GenBank ID:NP_001088948).

Phylogenetic analysis of RXRs from 17 species was performed using the amino acid sequences of DBDs and LBDs, respectively. The phylogenetic trees based on DBD (Figure 3A) and LBD (Figure 3B) sequences were different. The DBD tree included two main branches. One main branch only contained Vertebrata (*Danio rerio, Homo sapiens, Mus musculus, Xenopus laevis*) and the other main branch included three invertebrate classes. Within the invertebrate group, Insecta (*Aedes aegypti, Apis mellifera, Bombyx mori,* *Tenebrio* *molitor* *,* *Locusta* *migratoria* ) formed a clade and then united with the Crustacea (*Uca pugilator,* *Daphnia* *magna* *,* *Gecarcinus* *lateralis* ) clade. Together with the other mollusks ( *Biomphalariaglabrata* *, Lymnaea stagnalis,* *Reishia* *clavigera* *,* *N* *. lapillus* ), *C* *. farreri* formed the Mollusca clade. LBDs reflect the differences in ligand preference for RXRs among species [24,25]. As shown in Figure 3B, the LBDs of mollusks clustered as a clade, and CfRXR was included in the same main branch. Of the remaining groups, the Vertebrata branched and diverged, followed by the Crustacea. Lastly, the Insecta diverged into different branches. The mollusk LBDs were more similar to the vertebrate LBDs than they were to the crustacean LBDs. The branching depicted in the DBD tree resembled the accepted evolutionary history of these organisms, while the LBD tree showed some difference, which mirrored the different amino acid replacement rate between the two domains.

### CfRXRs mRNA expression profiles

**Figure 4:**
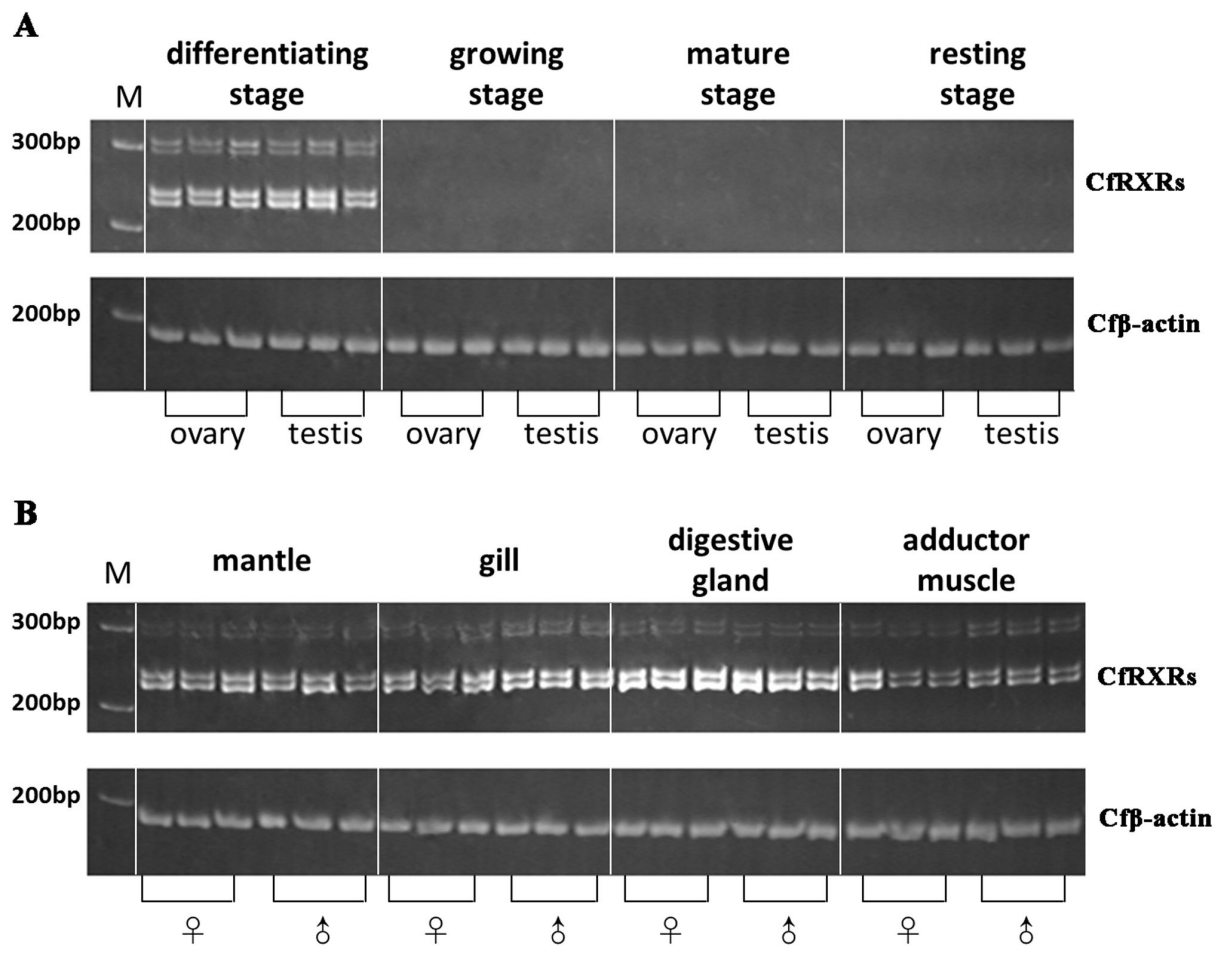
RT-PCR analysis of *CfRXR*s expression. Transcripts of the four *CfRXR* isoforms were detected at the differentiating stage of both ovaries and testes (A), and in mantle, gill, digestive gland, and adductor muscle in sexually mature male and female scallops (B). For each of the developing stage of gonad and other tissues, RT-PCR was performed in three individuals. On the gel showing RT-PCR products of *CfRXR*s, from top to bottom, the four bands in a single lane correspond to *CfRXRd*, *CfRXRc*, *CfRXRb*, and *CfRXRa*, respectively. The *β-actin* cDNA fragments (*Cfβ-actin*) were amplified as internal control.

To examine the expression levels of *CfRXR*s in scallop gonads during different developmental stages, total RNA samples from ovaries and testes at different gonadal stages were subjected to RT-PCR with a primer pair that could discriminate the four isoforms by the length of PCR products. For the four stages (differentiating, growth, mature and resting) examined, expression of all of the four isoforms were detected in both ovaries and testes during the differentiating stage, and that none of the isoforms was observed in the other three stages (Figure 4A). This result suggests that the *CfRXRs* may play a role in the regulation of germ cell differentiation. We also examined the expression of *CfRXR* mRNAs in other tissues of sexually mature male and female Zhikong scallops. Transcripts of the four isoforms were detected in all the tissues sampled, including mantle, gill, digestive gland, and adductor muscle (Figure 4B), which implied multiple biological functions of *CfRXRs*.

## Discussion

### Structure of CfRXRs

Four cDNAs corresponding to four isoforms of Zhikong scallop *RXR*, named *CfRXRa, CfRXRb, CfRXRc* and *CfRXRd*, were identified in this study. This is the first report of RXR isoforms in bivalves. The predicted proteins were identical with the exception of amino acid insertions/deletions located in the T-box. The three variants, termed T (+4), T (+20), and T (+24) contained insertions of 4, 20 and 24 amino acids, respectively. A unique match with only one scaffold in the Zhikong scallop genome database indicates that the four isoforms are derived from alternatively spliced mRNA from a single gene. A similar finding has been reported in two species of mollusks [11,18]. Two RXR variants, which differ by five amino acids in the T-box, have been found both in *N* *. lapillus* [18] and *T* *. clavigera* [11]. In insects, most RXR/USP isoforms differ in the A/B domain. They are generated through the use of different promoters and/or alternative splicing [12,13,26,27]. RXR isoforms differing in their LBDs have also been reported in *L* *. migratoria* [28] and *Blattella* *germanica* [8]. Unlike insect RXRs, the RXR/USP isoforms in crustaceans contain identical A/B domain sequences [16,17,29,30,31] but have differences in the T-box [15,17,29,31] or in the LBD [16,17,30,31]. Therefore, mollusks might have a mechanism for generating RXR diversity that is more similar to the crustacean mechanism than it is to the insect mechanism. As for the inserts position in the T-box of the RXR isoforms, there is difference between mollusks and crustaceans. The T-box has a conserved amino acid sequence (KREAVQEER). The insertion position in mollusks, including scallops and snails (Figure 1), is between residues A and V [11,18], but in crustaceans the insertion is between Q and E [15,17]. The T-box is responsible for the formation of RXR homodimer complexes in DR1 and for the formation of heterodimers when RXR occupies the site downstream of DR1 [32,33]. Although the ecdysteroid receptor (EcR) has been reported as the candidate partner of RXR in the mollusk *Lottia* *gigantean* [34], it is not known whether receptors other than EcR can dimerize with RXR. It is possible that T-boxes with different insertions can recognize different RXR partners and thus form different heterodimers. Meanwhile, for different species, the mechanisms of RXR isoforms generation are different. Most vertebrate and insect RXR isoforms differ in the A/B domain sequences, whereas their differences in crustaceans are in the C and E domains. For mollusks including gastropods and scallop, all the RXR isoforms identified by far are only different in the C domain.

### Phylogentic characterization of CfRXR

Differences in the LBDs of RXRs between mollusks, other invertebrates and vertebrate species were reflected in the phylogenetic tree shown in Figure 3B. RXRs from scallops and other mollusks clustered as a clade. This group was the sister group to that of the vertebrates, indicating that mollusk RXRs are more closely related to vertebrate RXRs than they are to the RXRs of other invertebrates. This may reflect functional conservation of RXRs between vertebrates and mollusks with respect to ligand-binding characteristics. The vertebrate RXR preferentially binds 9-cis RA [14], and 9-cis RA was found to be a ligand with high affinity for RXRs in the gastropods *T* *. clavigera* , *B. glabrata* *and* *N* *. lapillus* [10,18,35]. The above phylogenetic analysis, along with the conserved residues that interact with 9-cis RA (Figure 1), suggests that 9-cis RA is the candidate ligand for the *C* *. farreri* RXRs though further investigation needs to be conducted to verify it. In contrast to the LBDs, the DBDs of scallop and other mollusks are more closely related to the insect and crustacean DBDs, and an branch based on invertebrate and vertebrate DBDs was formed, respectively. These data suggest that the DBD and LBD of RXRs have experienced different selective pressures during the course of evolution.

### Expression patterns of CfRXRs

The expression of the four *C. farreri* *RXR* isoforms were only detected in the ovary and testis during the differentiating stage of the gonads, while in the other three stages examined (growth, mature and resting), transcripts of the four *CfRXR* isoforms were not found, suggesting the roles of *CfRXRs* in germ cell differentiation stage in both sexes. A similar result has been reported in a neogastropod. Mud snail *RXR* mRNA is expressed in coordination with sex differentiation, and both sexes expressed the highest levels of *RXR* mRNA during the sex differentiation stage [21]. It is still not known how RXR is involved in the biochemical signaling pathways of sex differentiation in mollusks. Sternberg [21] proposed a model whereby photoperiod and temperature, which are important for the timing of reproduction in mollusks [36], may induce repression of the RXR gene by a repressor element such as SMRT or NCoR, after the sex differentiation stage. For scallop, there might be similar RXRs expression regulation during sex development. Studying the effect of the factors on RXR regulation, such as hormonal, temperature, food supply or photoperiod, might provide more information to reveal the mechanism underlying RXR regulation and sex development in scallop.

Expression differences in *RXR* and *USP* isoforms have been reported in several species [8,11,12,15,17,27,37]. In *B. germanica, BgRXR-L* is expressed during early embryogenesis, whereas *BgRXR-S* is highly expressed in middle and late embryogenesis. In addition, *BgRXR-S* mRNA predominates in the fat body, whereas *BgRXR-L* mRNA is predominant in the adult ovary [8]. The transcriptional activity of TcRXR-2, which has a five-amino acid insertion in the T-box, is signiﬁcantly lower than that of TcRXR-1 in the snail *T* *. clavigera* [11]. These results imply that different isoforms of *RXR/USP* have different functions or transcriptional activities, while in scallop, no obvious changes in the expression level of the four *CfRXRs* was observed between the ovary or testis during the differentiation stage. Transcripts of the four isoforms were also detected in all the other tissues examined in sexually mature male and female individuals. Considering the role of the T-box, we propose that the four CfRXR isoforms which differ only in T-box, may form different homo/heterodimers and then bind to the DR1s of different target genes involved in the process of germ cell differentiation, and in different biological processes of other tissues in Zhikong scallops. Further research focusing on the expression regulation of the *CfRXR* isoforms during scallop gonad development and in different cell types will broaden our understanding of germ cell differentiation and RXR functions in bivalves.
